# Could Pyelonephritic Scarring Be Prevented by Anti-Inflammatory Treatment? An Experimental Model of Acute Pyelonephritis

**DOI:** 10.1155/2014/134940

**Published:** 2014-07-03

**Authors:** Elif Bahat Özdoğan, Tuğba Özdemir, Seçil Arslansoyu Çamlar, Mustafa İmamoğlu, Ümit Çobanoğlu, Bircan Sönmez, İlknur Tosun, İsmail Doğan

**Affiliations:** ^1^Department of Pediatrics, Division of Pediatric Nephrology, Faculty of Medicine, Karadeniz Technical University, 61080 Trabzon, Turkey; ^2^Department of Pediatrics, Faculty of Medicine, Karadeniz Technical University, 61080 Trabzon, Turkey; ^3^Department of Pediatric Surgery, Faculty of Medicine, Karadeniz Technical University, 61080 Trabzon, Turkey; ^4^Department of Pathology, Faculty of Medicine, Karadeniz Technical University, 61080 Trabzon, Turkey; ^5^Department of Nuclear Medicine, Faculty of Medicine, Karadeniz Technical University, 61080 Trabzon, Turkey; ^6^Department of Microbiology, Faculty of Medicine, Karadeniz Technical University, 61080 Trabzon, Turkey

## Abstract

*Objectives.* This study aimed to demonstrate if the addition of anti-inflammatory treatment to antibiotic therapy shows any superiority to the treatment with antibiotic only. *Methods.* Forty-nine Wistar rats were divided into 7 groups. Pyelonephritis was performed by *E. coli* injection to upper pole of kidneys except control group. Group 2 was not treated. Ceftriaxone, ketoprofen, “ceftriaxone + ketoprofen,” methylprednisolone, and “ceftriaxone + methylprednisolone” were given in the groups. The technetium-99m-dimercaptosuccinic acid scintigraphies were performed in 3rd day to detect pyelonephritis and 10th week to detect renal scarring. All kidneys were also histopathologically evaluated. *Results.* When 3rd day and 10th week scintigraphies were compared, initial 2.00 ± 0.30 point pyelonephritis score resulted in 0.71 ± 0.36 renal scar score in “ceftriaxone + ketoprofen” group (*P* = 0.039). Initial 2.00 ± 0.43 point pyelonephritis score resulted in 0.86 ± 0.26 renal scar score in “ceftriaxone + methylprednisolone” group (*P* = 0.041). Renal scar score was declined in “ceftriaxone + ketoprofen” group and “ceftriaxone + methylprednisolone” group compared with no-treatment group on 10th week of the study (*P* = 0.026, *P* = 0.044). On histopathological evaluation, it was seen that renal scar prevalence and expansion declined significantly in “ceftriaxone + ketoprofen and ceftriaxone + methylprednisolone” (*P* = 0.011, *P* = 0.023). *Conclusion.* It was evidenced that ceftriaxone treatment in combination with ketoprofen or methylprednisolone declined scar formation in scintigraphic and histopathologic examinations of the kidneys.

## 1. Introduction

Urinary tract infection (UTI) in infants and children is a relatively common problem, with potentially serious consequences.

Technetium-99m-dimercaptosuccinic acid (DMSA) renal scintigraphy is considered the most sensitive test for the diagnosis of renal involvement and the subsequent development of renal scarring [1, 2].

It may still cause renal scar formation in up to 40% of cases, leading to hypertension, proteinuria, and end-stage renal disease in children [3]. Most important role belongs to acute inflammatory response in scar generation [3, 4].

Various anti-inflammatory treatments were experimental in order to prevent scar generation due to the importance of host origin cytokine in inflammation. Therefore, ongoing research projects are underway to find an agent that can prevent renal scarring and subsequent complications. Inhibition of acute inflammation in experimental studies by steroids [3, 5], anti-inflammatory agents [6, 7], melatonin [8], pentoxifylline [9], vitamin A [10], vitamins A and E [11], vitamins C and E [12], vitamin E [13], mesenchymal stem cell [14], methylene blue [15], dapsone [16], ulinastatin [17], and montelukast [18] have been reported to reduce kidney damage after infection.

It was thought that both ketoprofen and methylprednisolone may block such mechanisms which give acute inflammatory response, at various stages to prevent renal scar generation. This study aimed to demonstrate if the anti-inflammatory treatment in combination with antibiotic treatment shows any superiority to antibiotic treatment alone.

## 2. Material and Methods

### 2.1. Animals

In this study, 49 Wistar rats weighing between 150 and 200 g were used. Animals were housed in specific pathogen-free conditions at room temperature (22°C ± 2°C) using a 12/12-hour light/dark cycle and provided with commercially available rat chow and tap water ad libitum. All of the rats were 8–10 weeks old. Karadeniz Technical University animal ethics board approved number is “02.370.”

### 2.2. Bacteria

The* Escherichia coli* strain UTI 36, isolated from a previous patient with confirmed acute pyelonephritis and phenotyped for the presence of P-fimbria and hemolysin production, was grown overnight on Luria Bertani (LB) agar. Before infection, a single colony of bacteria was inoculated onto LB broth and grown at 37°C with shaking to the stationary phase, after which the organisms were centrifuged and washed twice in phosphate-buffered saline. A solution containing approximately 5 × 10^9^ organisms/mL was prepared. Antibiotic sensitivities were assayed using Kirby-Bauer disks impregnated with ceftriaxone.

### 2.3. Experimental Infection

All animals were anesthetized by intramuscular injection of ketamine hydrochloride at 80 mg/kg (Ketalar, Parke-Davis). The kidney was exposed through a midline abdominal incision, and 0.1 mL of bacterial solution (5 × 10^9^ colony-forming units/mL) was then injected to upper pole.

### 2.4. Scintigraphic Imaging

**Figure 1:**
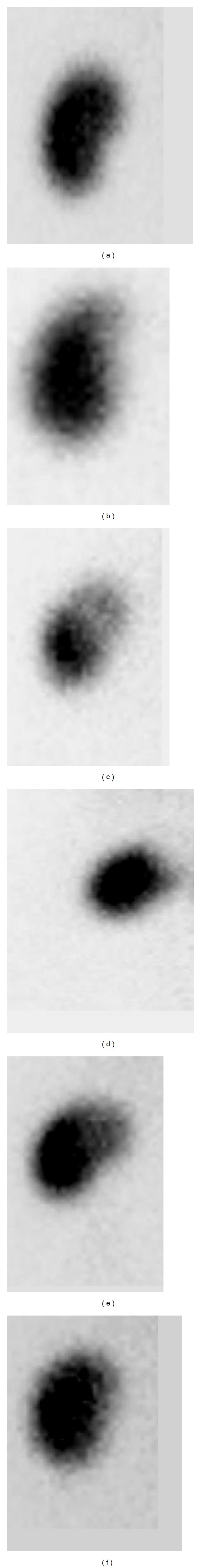
(a) DMSA kidney scintigraphy that indicates normal activity retention in the 3rd day in control group (pyelonephritis score: 0). (b)DMSA kidney scintigraphy in the 3rd day in ceftriaxone group (pyelonephritis score: 1). (c) DMSA kidney scintigraphy in the 3rd day in ceftriaxone-ketoprophen group (pyelonephritis score: 3). (d) DMSA kidney scintigraphy in the 10th week in no-treatment group (scar score: 2). (e) DMSA kidney scintigraphy in the 10th week in ceftriaxone group (scar score: 1). (f) DMSA kidney scintigraphy in the 10th week in Ceftriaxone − Ketoprophen Group (scar score: 0).

In the 3rd day (48–96th hours) of the study, the technetium-99m-dimercaptosuccinic acid (DMSA) renal scintigraphies of all rats including the control group were taken and the rats were classified according to the presence and expansion of pyelonephritis (Figures 1(a), 1(b), and 1(c)). Furthermore, the DMSA renal scintigraphies of all rats were taken a second time at the 10th week of the study and their kidneys were classified according to the presence and expansion of renal scarring (Figures 1(d), 1(e), and 1(f)).

Scar or pyelonephritis score was assessed using a renal damage score. Each renal unit was divided into three equal zones, and lesions were graded based on percent of affected cortex. Renal scars were each graded by DMSA scan from 0 to 3 according to the extent of pyelonephritic lesions of varying severity involvement as follows: 0, if no damage; 1, if less than 33% damage; 2, if between 33 and 66% damage; 3, if more than 66% damage [5].

### 2.5. Experimental Groups

The rats were divided equally into seven groups each containing seven rats. In control group, sham operated group (Group 1) consisted of healthy rats. Pyelonephritis was induced by injection of* E. coli* to other rats as mentioned above. In no-treatment group (Group 2), the rats had pyelonephritis but did not receive any treatment. Treatments began 72 hours after bacterial inoculation in the other groups. The rats in ceftriaxone group (Group 3) were treated only with ceftriaxone (i.m) at a dose of 50 mg/kg once daily for 10 days. In ketoprofen group (Group 4), ketoprofen injections were done at a dose of 2 mg/kg for 3 days. Two rats with no indication about infection in 3rd day scintigraphic examination in group 4 were excluded from the study. The rats in “ceftriaxone plus ketoprofen” group (Group 5) were treated with 50 mg/kg ceftriaxone for 10 days and 2 mg/kg ketoprofen for 3 days before 30 minutes of ceftriaxone administration. The rats in methylprednisolone group (Group 6) were given 30 mg/kg methylprednisolone for 3 days. The rats in “ceftriaxone plus methylprednisolone” group (Group 7) were given 50 mg/kg ceftriaxone for 10 days and 30 mg/kg methylprednisolone for 3 days before 30 minutes of ceftriaxone administration.

### 2.6. Histopathologic Examination

After routine processing, each half renal unit was divided into three equal zones (upper, middle, and lower), and five sections were obtained from anterior zone and five from posterior zone. The sections were obtained through the renal cortex to the collecting system. The sections were stained with hematoxylin-eosin and Masson's trichrome. A pathologist, who was unaware of the group designations, evaluated the specimens. Two main histopathologic changes were regarded as microscopic criteria: the inflammatory response (interstitial mononuclear inflammatory cell infiltration) and cicatrization (interstitial fibrosis-tubular atrophy). These changes were scored semiquantitatively for comparison purposes. The two criteria were each graded from 0 to 3 according to the extent of parenchymal involvement: 0, if none was involved; 1, if less than 5% of the parenchyma was involved; 2, if more than 5% and less than 10% of the parenchyma was involved; and 3, if more than 10% of the parenchyma was involved [8].

### 2.7. Sacrifice of Animals

The rats in were sacrificed under anesthesia, ten weeks after bacterial inoculation to determine the extent of renal scar formation.

### 2.8. Statistical Analysis

SPSS (statistical package for social science) for Windows was used for statistical analyses. *P* < 0.05 was regarded as significant. DMSA scintigraphic scores of 3rd day and 10th week are compared with using “Wilcoxon” test. No-treatment group and other groups were compared with “Kruskal Wallis” test on the base of 10th week DMSA scintigraphic results. No-treatment group and other groups were compared with “Mann Whitney U” test on the base of histopathological results.

## 3. Results

Table 1 shows the result of DMSA kidney scintigraphy (Figures 1(a)–1(f)) of the 3rd day pyelonephritic scores and of the 10th week renal scarring scores.

When the rats were evaluated in terms of the DMSA renal scintigraphy findings, it was found that in the no-treatment group the pyelonephritic involvement score decreased from 2.00 ± 0.21 in the 3th day to 1.71 ± 0.18 scar score at the end of the 10th week. (*P* = 0.157).

In the group which received only ceftriaxone treatment, the pyelonephritic involvement score was found 1.43 ± 0.29 in 3th day and 1.00 ± 0.30 renal scar score at the end of the 10th week. (*P* = 0.083).

In the group which received “ceftriaxone plus ketoprofen” treatment, the pyelonephritic involvement score decreased from 2.00 ± 0.30 in the 3th day to 0.71 ± 0.36 renal scar score at the end of the 10th week. (*P* = 0.039).

In the group which received “ceftriaxone plus methylprednisolone” treatment, the pyelonephritic involvement score decreased from 2.00 ± 0.43 in the 3rd day to 0.86 ± 0.26 renal scar score at the end of the 10th week (*P* = 0.041).

Table 2 shows no-treatment group compared with other groups on the base of the 10th week DMSA scintigraphic results.

Scar scores were low in the “ceftriaxone plus ketoprofen” and “ceftriaxone plus methylprednisolone” groups when compared with no-treatment group (*P* = 0.026, *P* = 0.044, resp.) as shown in Table 2. Scar score in ceftriaxone treated group was not significant compared to no-treatment group. (*P* = 0.080).

**Figure 2:**
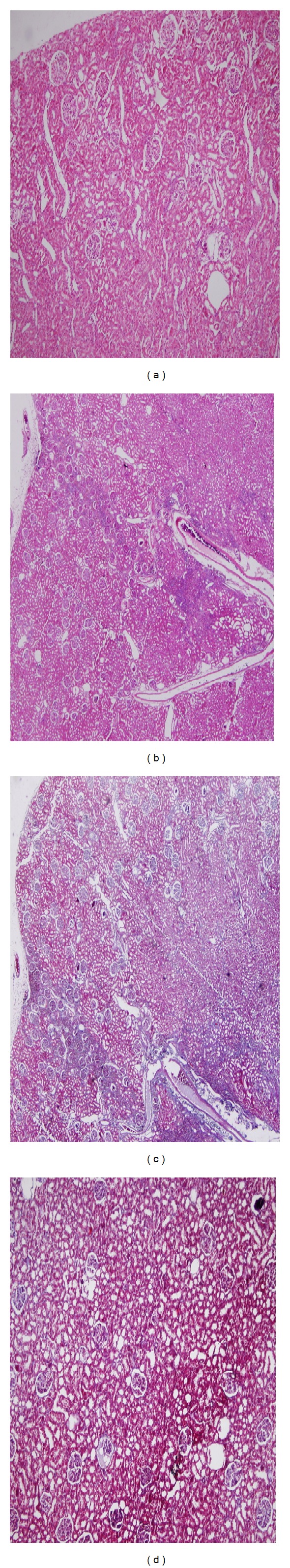
(a) Control group kidney tissue which does not indicate any pathological finding in renal parenchyma (H.E ×100). (b) 2nd degree inflammation in renal parenchyma, no-treatment group (HE ×40). (c) 2nd degree scar in renal parenchyma, no-treatment group (M.T ×40). (d) Ceftriaxone-ketoprofen group kidney tissue which does not indicate any pathological findings in renal parenchyma.

After the histopathological evaluation, when the no-treatment group was compared with other groups in terms of the presence and expansion of renal scars, a statistically significant decrease was observed in the presence and expansion of renal scars in the “ceftriaxone plus ketoprofen” and “ceftriaxone plus methylprednisolone” groups (*P* = 0.011, *P* = 0.023, resp.) (Figures 2(a)–2(d)) (Table 3). Only ceftriaxone treated group is not significantly different compared to no-treatment group (*P* = 0.053).

## 4. Discussion

Pyelonephritis, an acute infectious disease of kidney parenchyma, now being considered common and serious, bacterial infection that occurs in infancy and early childhood. Renal scarring is a frequent outcome of acute pyelonephritis in children, reported in up to 65% of patients with pyelonephritis. The development of scars in early life, particularly in patients with VUR, has been correlated with the development of hypertension [19], preeclampsia, proteinuria, renal insufficiency, and end-stage renal disease. Of all patients with endstage renal disease, chronic pyelonephritis has been reportedly the cause in 10 to 25% of children, 7–17% in the world, and 23.6% in our country. Antibiotic treatment is important but not minimizing renal damage and scarring alone.

*Escherichia coli* is the most common organism present up to 80% in UTI as we used in our study, although other enteric organisms such as* Klebsiella* spp. and enterococci, as well as* Staphylococci*, have been identified [20]. Bacterial inoculation in the tissue, ischemia reperfusion damage, and lysosomal lytic enzyme retention cause renal scar by means of endoxines, cytokines, and chimiotaxy [4]. Cytokines play a major role in renal scar formation [3].

It has been reported that renal damage after acute pyelonephritis is more closely related to the extent of the inflammatory process associated with infection than the actual bacterial growth in kidney [21, 22]. The inflammatory response following bacterial inoculation is characterized by recruitment of activated neutrophils and lymphocytes to renal tissue and the release of antibacterial substances such as free radical species and lysosomal enzymes [23]. Therefore, anti-inflammatory treatment is believed to be effective in preventing renal scarring. [5–7, 24, 25]. Glucocorticoids are widely used for the suppression of inflammation [26].

Previous experimental studies reported that technetium-99m-dimercaptosuccinic acid (Tc-99m-DMSA) renal scintigraphy is highly sensitive and reliable for the detection of acute pyelonephritis when performed during the acute phase of infection and renal scaring when performed after recovery. DMSA scintigraphy is considered the most sensitive test for the diagnosis of renal involvement and the subsequent development of renal scarring [1, 2]. The renal cortical changes are acceptably detected by Tc-99m-DMSA renal scintigraphy. In our study, DMSA scan findings were comparable with histopathological results.

Recent experimental studies demonstrate that oxygen-free radical scavengers and antioxidants can reduce tissue damage and renal scaring during acute and chronic pyelonephritis. Antioxidant vitamins [3, 9] increase tissue protection from oxidative stress. Bennett et al. showed that vitamins A and E suppressed renal inflammation in pyelonephritis [11]. Kanter et al. showed that vitamin C treatment alone or with vitamin A may prevent endotoxin-induced renal damage [27]. Imamoğlu et al. measured the level of tissue malondialdehyde in an experimental pyelonephritis model in rats and showed that combined with antibiotics and melatonin it may decrease the inflammation [8]. From the report by Yagmurlu et al., it is showed that anticytokine activity of pentoxifylline could be the other mechanism for the prevention of renal scarring due to pyelonephritis, though this study did not include cytokine measurements [9]. The preventive effect of dapsone which has a scavenging activity on active oxygen species on renal scarring was found to effectively prevent renal scarring by Mochida et al. [16]. Caffeic acid phenethyl ester (an active component of propolis from honeybee hives, which has antioxidant, anti-inflammatory, and antibacterial properties) administration reduced significantly decreased* E. coli*-induced lipid peroxidation as showed by Celik et al. [20]. Cyclophosphamide due to effect of neutropenia and inhibition leukocyte migration of colchicine was used to leukocyte modulation and found that they can prevent renal scarring by Matsumoto et al. But it is not useful because of serious side effects of this agent [28]. In a study by Patel et al. the degree of renal dysfunction and inflammation caused by ischemia-reperfusion was significantly reduced in 5-lipoxygenase knockout mice as compared to wild type mice. Moreover, administration of 5-lipoxygenase inhibitor before ischemia-reperfusion significantly reduced the degree of renal dysfunction and injury [29]. Mesenchymal stem cells (rMSC) were shown to have therapeutic value in alleviating pyelonephritis-associated histopathologic changes in rats [14]. Nevertheless, in real clinical practice, the beginning of the infectious process is silent and cannot be used for antioxidant treatment [13]. Haraoka et al. confirmed the active role of inflammation in renal scarring by demonstrating that prednisolone was sufficient to prevent renal scar formation in rats with APN receiving delayed antibiotics treatment [22]. Surgery performed vezicouretheral reflu on pigs was created as an experimental pyelonephritis model. Pohl et al. investigated the effect of preventing the renal scarring of oral prednisolone [5]. Also, in a study by Sharifian et al. it was concluded that the administration of dexamethasone could possibly prevent the formation of kidney scar [3]. As similar to our study, combined antibiotic with ibuprofen, an inhibitor of cyclooxygenase, and neutrophil chemotaxis was expected to decrease renal scar formation resulting from inflammation by Huang et al. [7].

In this study, it was found that ceftriaxone treatment in combination with ketoprofen or methylprednisolone decreased renal scar development in pyelonephritis. Although there was some decrease in scar expanse compared to the expansion of the pyelonephritic involvement in only ceftriaxone treatment group, this value was not statistically significant. The rats which received no treatment developed scars whose expansion was proportionate to the expansion of the pyelonephritic involvement.

Compared to previous studies, in all infected rats, pyelonephritis was verified via DMSA and scar verified both DMSA and histopathologic examination. In accordance with the aforementioned studies, our results show that adding ketoprofen or methylprednisolone to ceftriaxone treatment declines scar formation in experimental pyelonephritis. Ceftriaxone treatment is not effective as ceftriaxone plus anti-inflammatory treatment to prevent renal scarring.

Ketoprofen is a nonsteroidal anti-inflammatory drug used for six-month and older infants as analgesic and antipyretic drug approved by Food and Drug Administration and promising good results when used as anti-inflammatory therapy to prevent renal scarring for febrile pyelonephritic children as well as antipyretic effect.

Our study is the first study that showed scar by both DMSA and histopathological examination. The results were consistent with the literature. We hope this information will have potential use in minimizing the renal scarring associated with pyelonephritis in children.

## 5. Conclusion

Our results provide ceftriaxone with ketoprofen OR methylprednisolone can effectively decrease cellular damage and prevent long-term complications in acute pyelonephritis.

In conclusion, the study showed both histopathologically and with DMSA renal scintigraphy in an experimental pyelonephritis model on rats that the addition of ketoprofen or methylprednisolone to ceftriaxone treatment decreases scar development. The study is the first in the literature in which pyelonephritis was proven by DMSA and pyelonephritic scar was shown both histopathologically and with DMSA. There are few studies on this topic in the literature and our findings are congruent with the literature.

Given the fact that the most common causes of pediatric end stage renal disease in Turkey that develop renal scars are mainly pyelonephritis, the importance of studies and attempts towards the prevention of scars in pyelonephritis is apparent. According to the findings of the study, the addition of ketoprofen or methylprednisolone to ceftriaxone treatment is seen as an intervention that will contribute to the realization of this aim. It is hoped that this study will pioneer the clinical studies to be conducted for the aim of preventing scar development.

## Figures and Tables

**Table 1:** Results of DMSA kidney scintigraphy comparison of the groups between themselves with pyelonephritis score in the 3rd day and with scar score of DMSA scintigraphy in the 10th week.

Groups	PN score in 3rd day	Scar score in 10th week	*P*
Control	0 ± 0	0 ± 0	1.00
No-treatment	2.00 ± 0.21	1.71 ± 0.18	0.157
Ctx	1.43 ± 0.29	1.00 ± 0.30	0.083
Ktp	2.00 ± 0.44	1.20 ± 0.20	0.157
Ctx + Ktp	2.00 ± 0.30	0.71 ± 0.36	**0.039**
Mp	1.71 ± 0.47	1.14 ± 0.26	0.102
Ctx + Mp	2.00 ± 0.43	0.86 ± 0.26	**0.041**

**Table 2:** Results of DMSA kidney scintigraphy in 10th week no-treatment group and other groups were compared.

Groups	Scar score in 10th week	*P*
Control	0 ± 0	
No-treatment	1.71 ± 0.18	
Ctx	1.00 ± 0.30	0.080
Ktp	1.20 ± 0.20	0.093
Ctx + Ktp	0.71 ± 0.36	**0.026**
Mp	1.14 ± 0.26	0.100
Ctx + Mp	0.86 ± 0.26	**0.044**

**Table 3:** Results of histopathological renal scar assessment. No-treatment group and other groups were compared.

Groups		*P*
Control	0	
No-treatment	1.86 ± 0.40	
Ctx	0.86 ± 0.26	0.053
Ktp	1.20 ± 0.49	0.221
Ctx + Ktp	0.57 ± 0.20	**0.011**
Mp	2.14 ± 0.67	0.946
Ctx + Mp	0.57 ± 0.29	**0.023**
